# Single‐Molecule Electrical Profiling of Peptides and Proteins

**DOI:** 10.1002/advs.202401877

**Published:** 2024-04-19

**Authors:** Hongyu Ju, Li Cheng, Mengmeng Li, Kunrong Mei, Suhang He, Chuancheng Jia, Xuefeng Guo

**Affiliations:** ^1^ School of Pharmaceutical Science and Technology Tianjin University Tianjin 300072 P. R. China; ^2^ Center of Single‐Molecule Sciences Institute of Modern Optics Frontiers Science Center for New Organic Matter Tianjin Key Laboratory of Microscale Optical Information Science and Technology College of Electronic Information and Optical Engineering Nankai University Tianjin 300350 P. R. China; ^3^ Beijing National Laboratory for Molecular Sciences National Biomedical Imaging Center College of Chemistry and Molecular Engineering Peking University Beijing 100871 P. R. China

**Keywords:** nanopores, protein sequencing, silicon nanowires, single‐molecule detection, single‐walled carbon nanotubes

## Abstract

In recent decades, there has been a significant increase in the application of single‐molecule electrical analysis platforms in studying proteins and peptides. These advanced analysis methods have the potential for deep investigation of enzymatic working mechanisms and accurate monitoring of dynamic changes in protein configurations, which are often challenging to achieve in ensemble measurements. In this work, the prominent research progress in peptide and protein‐related studies are surveyed using electronic devices with single‐molecule/single‐event sensitivity, including single‐molecule junctions, single‐molecule field‐effect transistors, and nanopores. In particular, the successful commercial application of nanopores in DNA sequencing has made it one of the most promising techniques in protein sequencing at the single‐molecule level. From single peptides to protein complexes, the correlation between their electrical characteristics, structures, and biological functions is gradually being established. This enables to distinguish different molecular configurations of these biomacromolecules through real‐time electrical monitoring of their life activities, significantly improving the understanding of the mechanisms underlying various life processes.

## Graphical abstract

This work presents a timely overview of the prominent research progress in peptide and protein‐related studies through single‐molecule electrical techniques, including single‐molecule junction, single‐molecule field‐effect transistor (FET), and nanopore. The charge transport mechanism, detection of biological interactions, visualization of dynamic biological processes, and single‐molecule accurate sequencing are discussed in depth, respectively.

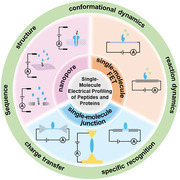

## 1 Introduction

Proteins and peptides are indispensable players in various fundamental life processes, such as enzymatic reactions, substance and energy transport, and cytoskeleton construction. Over the years, ensemble studies employing techniques like mass spectrometry,^[^ ^1^ ^]^ cryo‐electron microscopy,^[^ ^2^ ^]^ nuclear magnetic resonance spectroscopy (NMR),^[^ ^3^ ^]^ single crystal X‐ray diffraction (SCD),^[^ ^4^ ^]^ fluorescence resonance energy transfer (FRET),^[^ ^5^ ^]^ and fluorescence polarization^[^ ^6^ ^]^ have provided extensive information in spatial configurations, sequence details, and activities of proteins. However, real‐time monitoring of their dynamic processes at the single‐molecule and single‐step level remains a formidable challenge for these techniques. In general, molecules with different structures and spatial arrangements display distinct charge transport characteristics. Taking advantage of this property, single‐molecule electrical analysis techniques, such as nanopore, single‐molecule junction (SMJ), and single‐molecule field‐effect transistor (FET), have demonstrated unique advantages in real‐time monitoring in‐situ changes in molecular structure through current fluctuations. This is particularly attractive for studying dynamic biological processes, potentially enabling the identification of individual steps in a process, e.g., enzymatic reactions, and the capture of intermediates and transition states within it.^[^ ^7^ ^]^

In this review, we begin with a concise introduction to the operating principles of single‐molecule electrical analysis techniques. Then, we delve into their recent applications in examining the structures and functions of peptides and proteins in detail. Techniques such as SMJ and single‐molecule FET are employed to investigate the charge transport through amino acids and peptides‐ the building blocks of protein. Following this, a comprehensive analysis of larger, more complex proteins is presented, covering aspects such as conductance, sequence information, and spatial structure. Special attention is given to dynamic studies, with a particular focus on conformational dynamics and reaction dynamics, as illustrated with the examples of polymerase and protein kinase, therefore highlighting the unique advantages of these techniques (including nanopores) in protein sequencing and structural characterization based on the distinctive electrical characteristics of the analytes. These research findings demonstrate the promising applications of these techniques in disease diagnostics and drug discovery. Finally, the review concludes with an outlook on future developments and potential challenges in the research area. We believe that these single‐molecule electrical testing techniques will significantly contribute to advancing our understanding of the structural characteristics and dynamic behaviors of peptides and proteins, thereby deepening insights into their biological functions and underlying principles.

## 2 Single‐Molecule Electrical Testing Techniques

So far, a variety of single‐molecule electrical techniques have been designed and constructed for the purpose of characterizing proteins, including those based on SMJs, single‐molecule FETs, and nanopore techniques.^[^ ^8^ ^]^ These techniques demonstrate exceptional proficiency in providing highly sensitive, real‐time, and in‐situ electrical signals closely associated with biomacromolecular processes.

**Figure 1:**
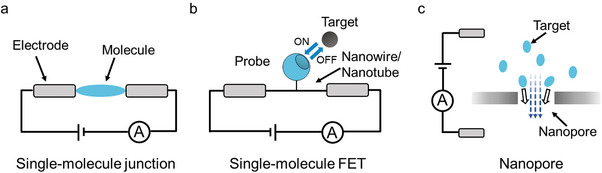
Schematic presentation of simplified single‐molecule electrical techniques. a) Single‐molecule junction. b) Single‐molecule field‐effect transistor. c) Nanopore system.

A single‐molecule junction, in particular, is a structure in which an analyte or functional molecule bridges between two nanoelectrodes (**Figure** **1**a). Based on structural differences and electrode materials, SMJ can be divided into several categories: scanning tunneling microscopy break junction (STM‐BJ),^[^ ^9^ ^]^ mechanically controllable break junction (MCBJ),^[^ ^10^ ^]^ graphene‐molecule‐graphene single‐molecule junction (GMG‐SMJ),^[^ ^11^ ^]^ and single‐walled carbon nanotube single‐molecule junction (SWCNT‐SMJ).^[^ ^12^ ^]^ Both STM‐BJ and MCBJ operate on a similar principle: one metal electrode repeatedly makes contact with and then retracts from another electrode. During this process, a single molecule transiently connects with the electrodes, forming a so‐called molecular junction within the nanogap. This junction breaks when the gap is larger than the length of the molecule. Since numerous junctions can be formed and broken in repeated experiments, these two techniques are well‐suited for statistical analysis with high efficiency. The molecule junction in the GMG‐SMJ and SWCNT‐SMJ techniques, on the other hand, relies on the fixed and stable covalent bonds with two functionalized electrodes, making them better suited for long‐time measurements with good resolution and controllability.^[^ ^13^ ^]^

In a single‐molecule FET device, the functional molecule is typically adsorbed onto a low‐dimensional nanomaterial that serves as a bridge between the two electrodes (Figure 1b). SWCNT and silicon nanowires (SiNW) are the most commonly chosen materials due to the advantages of good biocompatibility, comparable size with biomolecules, high sensitivity to charge changes, and distant transmission along the 1D channel.^[^ ^14^ ^]^ The functional molecule can be adsorbed onto the bridge via either covalent bonds or non‐covalent interactions.^[^ ^15^ ^]^ Unlike the conventional planar gate electrode, the adsorbed (functional) small molecules play the role of a local gate through which the potential at the FET channel‐electrolyte interface can be conveniently regulated.^[^ ^16^ ^]^ Although other free‐charged molecules (or ions) exist in the solution, they can be electrostatically screened by the electric double layer at the electrode surface when electrolyte‐contained buffer solutions are used.^[^ ^17^ ^]^ This screening effect is associated with the electrolyte concentration, where a higher concentration results in a smaller Debye length (λ_D_). When the distance between device surfaces and free charges is >λ_D_ (typically ≈ 1 nm in physiological fluids), the electrostatic interaction of free charges on the FET channel becomes negligible.^[^ ^18^ ^]^

The primary structure of a nanopore system is a nano‐scale channel embedded in a membrane material, with two chambers filled with electrolytes on either side of the membrane (Figure 1c).^[^ ^19^ ^]^ The translocation of particles from one chamber to the other can disrupt the equilibrium state of ion flow within the nanopore, consequently triggering a unique current block due to volume exclusion and non‐covalent interactions.^[^ ^20^ ^]^ The resulting amplitude, frequency, and duration of the blockade currents collectively form a distinctive electrical fingerprint of the analyte, enabling molecular recognition at the single‐molecule level, e.g. DNA and protein sequencing. The mainstream nanopore systems are broadly categorized into two major groups based on the channel‐building materials: biological nanopores and solid‐state nanopores. Biological nanopores are based on natural pore‐forming proteins embedded in a membrane lipid bilayer.^[^ ^21^ ^]^ They not only have well‐defined and reproducible size and structure but are also compatible with various surface modifications, thereby possessing broad application potentials in protein characterizations.^[^ ^22^ ^]^ Compared to biological nanopores, solid‐state nanopores are generally more structurally and chemically stable, exhibit superior structural design flexibility, and are more conducive to large‐scale integration. While silicon composite films (SiN and SiO_2_) remain the most popular choice for constructing solid‐state nanopores, other materials such as HfO_2_, ZnO, and Al_2_O_3_ have also proven to be promising candidates.^[^ ^23^ ^]^ Beyond these two main types of nanopores, various other strategies for nanopore construction have been explored. Hybrid nanopores, for instance, involve the insertion of channel proteins into a solid‐state matrix.^[^ ^19^, ^24^ ^]^ Nanopores constructed on 2D materials with sub‐nanometer thickness, including graphene,^[^ ^25^ ^]^ BN,^[^ ^26^ ^]^ and MoS_2_,^[^ ^27^ ^]^ have demonstrated theoretical atomic‐scale spatial resolution and attracted considerable attention. In addition, the programmed assembly of DNA single strands, known as the DNA origami technique, has been explored for the bottom‐up fabrication of nanopores.^[^ ^28^ ^]^

In general, these techniques all rely on electrical currents as signals for analysis. However, when it comes to characterizing the properties of individual molecules, such as proteins, they vary significantly in their methodologies, advantages, and focuses.^[^ ^29^ ^]^ For both break junction and fixed junction techniques, the circuits are established via SMJs, where the current level reflects the charge transport characteristics of the molecule being analyzed. The difference is how data is gathered. In break junction measurements, countless junctions are established and broken. Although all signals originate from single molecules, they are unlikely to come from the same molecule each time. Therefore, molecular conductance obtained with this method represents a statistically significant average, making the results more suitable for comparison between different molecules. In contrast, fixed SMJ and FET devices immobilize molecules between electrodes, ensuring that signals consistently originate from the same molecule. This setup is particularly adept at capturing the changes in molecular structure over time, making it powerful for dynamic analysis and mechanistic studies. However, the obtained molecular conductance may represent only one or a few possibilities of the molecule and is more random compared to that obtained from break junction techniques. Nanopore techniques, on the other side, are mechanistically very different from the SMJ devices. Nanopore techniques measure current fluctuations resulting from ion flow through a nanopore, which changes as the molecule to be tested passes through. Therefore, the current blockade mainly depends on the size of the molecule rather than its charge transport characteristics. Therefore, the technique is suitable for molecular recognition, differentiation of functional groups, and sequencing purposes, rather than for acquiring single‐molecule conductance.

## 3 Single‐Molecule Analysis of Amino Acids and Peptides

**Figure 2:**
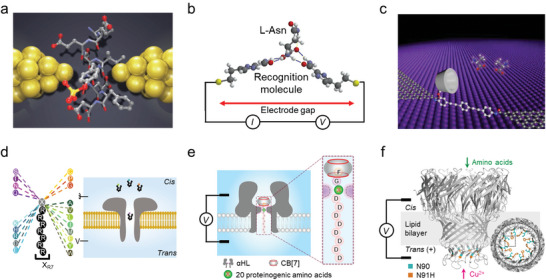
Electrical characterizations of single amino acids. Single‐molecule electrical testing of amino acid molecules using a) MCBJ with bare Au electrodes, b) STM‐BJ with functionalized Au electrodes, c) GMG‐SMJ upon host‐guest complexation, d) unfunctionalized nanopore, e) CB[7]‐functionalized and f) Cu^2+^‐functionalized nanopores. a) Reproduced with permission.^[^ ^10b^ ^]^ Copyright 2014, Springer Nature. b) Reproduced with permission.^[^ ^30^ ^]^ Copyright 2014, Springer Nature. c) Reproduced under the terms of the CC‐BY‐NC license.^[^ ^11b^ ^]^ Copyright 2021, The authors, published by American Association for the Advancement of Science. d) Reproduced with permission.^[^ ^31^ ^]^ Copyright 2019, Springer Nature. e) Reproduced with permission.^[^ ^32^ ^]^ Copyright 2023, Springer Nature. f) Reproduced under the terms of the CC‐BY license.^[^ ^33^ ^]^ Copyright 2024, the authors, published by Springer Nature.

Amino acids are fundamental building blocks of peptides and proteins. The efficient detection and characterization of amino acids are of critical importance to proteomics, pharmaceutics, nanobiotechnology, and supramolecular chemistry. In an MCBJ setup, the movement of an amino acid through the nanogap (with a size of 0.7 or 0.55 nm) between the two electrodes, driven by stochastic Brownian motion, can generate a conductance‐time profile composed of two factors: the maximum current *I*_p_ and the duration time *t*_d_ (**Figure** **2**a).^[^ ^10b^ ^]^ By analyzing the current‐time plots, 12 different amino acids have been identified. Specifically, a tyrosine molecule or tyrosine‐containing peptide can be discriminated from its phosphorylated variant.

In general, molecular recognition in electrical testing mainly relies on the intrinsic electrical properties of the analyte. The resolution and accuracy can be further improved by introducing specific intermolecular interactions, such as π‐π stacking and hydrogen bonding. For example, by covering the STM electrodes with a layer of recognition molecules, a chemically well‐defined functional surface (thickness of ≈2 nm) was fabricated (Figure 2b).^[^ ^30^ ^]^ Amino acids can be specifically captured and recognized via hydrogen bonding with the molecular layer and produce a higher current state with a duration time on the order of 0.2 s, allowing the identification of the d/l enantiomers, methylated amino acids, and isobaric isomers. In another case, a supramolecule host molecule (permethylated‐β‐cyclodextrin, PM‐β‐CD) was modified on the GMG‐SMJ molecular junction, thereby allowing the analytes, such as amino acids, to be captured and measured through the non‐covalent host‐guest complexation (Figure 2c).^[^ ^11b^ ^]^ With this design, natural amino acids and their enantiomers, and even different charge states of an amino acid were proved to be distinguishable. The fingerprint electrical signals, rather than solution properties, of a single amino acid with different structures and chirality, provide a route for highly sensitive detection and chiral recognition, and thus a better understanding of physiological functions in biological systems.

With nanopore technology, a strategy has been devised by chemically linking a proteinogenic amino acid (X) to a short polycationic carrier, arginine heptapeptide (R7), termed X_R7_ (Figure 2d).^[^ ^31^ ^]^ The blockade current and duration time measured on the resulting octapeptide can be used to differentiate the type of X. With this strategy, 13 out of the 20 amino acids were identified, and the remaining 7 can be divided into two groups due to the similarity of their signals. In another study, researchers have modified the nanopore portal with the synthetic host molecule cucurbit[7]uril (CB[7]). The modification significantly enhances the sensitivity of the method for all 20 amino acids by introducing additional van der Waals interactions with them (Figure 2e).^[^ ^32^ ^]^ Specifically, for the peptide FGXD_8_ (F, phenylalanine; G, glycine; D, aspartic acid), the interaction between CB[7] and FG moiety slows down the translocation of the peptide, resulting in improved signal resolution.

Accurately identifying standalone amino acids is crucial for protein degradation‐based sequencing methods. In a very recent study, a *Mycobacterium smegmatis* porin A (MspA) nanopore has been functionalized with copper ions by forming a Cu^2+^‐histidine complex with the N91H substitution in the constriction region (Figure 2f).^[^ ^33^ ^]^ As individual amino acid passes through the region, its interactions with the Cu^2+^ complex result in well‐distinguishable current blockade signals. This allows the identification of all 20 proteinogenic amino acids with a validation accuracy of 99.1%. A similar approach was employed in an earlier study using a Ni^2+^‐maleimido‐C3‐nitrilotriacetic acid (Ni‐NTA) functionalized MspA nanopore, 20 amino acids were differentiated with a general accuracy of 98.6%.^[^ ^34^ ^]^

Post‐translational modifications (PTMs), including phosphorylation, glycosylation, methylation, and acetylation, enhance the functional diversity of peptides and proteins.^[^ ^35^ ^]^ However, these modifications make the sequencing of proteins and peptides even more challenging, as they can no longer be directly interpreted from genomic information. A recent study demonstrated the detection of O‐glycosylation in a bipolar model peptide by using wild‐type Fragaceatoxin C (FraC) nanopores.^[^ ^36^ ^]^ In addition, thioredoxin (Trx) variants with different phosphorylation sites near the C‐terminal were successfully distinguished using α‐hemolysin (α‐HL) nanopores.^[^ ^37^ ^]^ The α‐HL nanopore has also been employed to detect post‐translational modifications deep within a lengthy peptide, where the translocation is propelled by electroosmotic flow rather than electrophoretic force.^[^ ^38^ ^]^ Moreover, the discrimination of one or two closely positioned phosphorylation sites within a 25‐amino‐acid peptide can be achieved with 95% accuracy through translocation controlled by a DNA motor enzyme.^[^ ^39^ ^]^ These studies demonstrate the promising application of nanopore techniques in peptide sequencing and PTMs detection.

**Figure 3:**
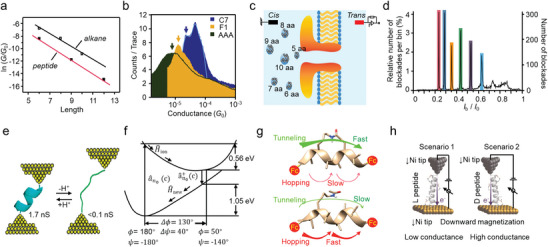
Electrical characterizations of single peptide. a) Relationship between conductance and molecular length of short peptides and alkanes with thiol terminal groups. Reproduced with permission.^[^ ^40^ ^]^ Copyright 2004, American Chemical Society. b) Conductance measurements of trialanine, amino‐octanoic acid, and 5‐(alanylamino)pentanoic acid. Reproduced with permission.^[^ ^41^ ^]^ Copyright 2018, American Chemical Society. c) Schematic illustration of the detection of homopolymeric peptides of different lengths with a nanopore, and d) the corresponding statistical analysis of output signals. Reproduced under the terms of the CC‐BY license.^[^ ^43^ ^]^ Copyright 2018, The Authors, published by Springer Nature. e) pH‐controlled interconversion between the folded and stretched conformations of peptides. Reproduced with permission.^[^ ^47a^ ^]^ Copyright 2011, American Chemical Society. f) Potential surface for charge transport versus the angular twist between neighboring amino acids. Reproduced with permission.^[^ ^48^ ^]^ Copyright 2007, WILEY‐VCH Verlag GmbH & Co. KGaA, Weinheim. g) Charge transport through constrained (upper) and linear (lower) peptides. Reproduced with permission.^[^ ^50^ ^]^ Copyright 2018, American Chemical Society. h) Conductance measurements of α‐peptide enantiomers on an STM‐BJ setup. Reproduced with permission.^[^ ^53^ ^]^ Copyright 2016, WILEY‐VCH Verlag GmbH & Co. KGaA, Weinheim.

A peptide is a chain molecule consisting of two or more amino acids that are linked through amide bonds. Charge transport along a peptide chain is influenced by the length, sequence, conformation, as well as dipole orientation of the peptide. Length‐dependent measurements through STM‐BJ and MCBJ show that charge transport through short‐stretched peptides is dominated by the tunneling mechanism, which exhibits a discernible dependence on molecular length with a decay constant of *β* = 0.87 ± 0.7 Å^−1^ (**Figure** **3**a).^[^ ^40^ ^]^ Furthermore, to eliminate the effects of non‐native linkers or arrangements in peptide measurements, the binding ability of amine and carboxyl groups with electrodes provides an opportunity for direct electrical measurements of the native transport behavior of the peptide backbone.^[^ ^8a^ ^]^ Conductance comparison among trialanine (AAA), 5‐(alanylamino)pentanoic acid (F1), and amino‐octanoic acid (C7) demonstrated that peptide was even more insulating than the alkanes (Figure 3b).^[^ ^41^ ^]^ The low conductivity of peptide has been attributed to the large molecular dipole, which reduces the energy of the frontier orbitals relative to the Fermi energy, and the tightly bound electrical states at the peptide bond that weaken the electronic coupling to the backbone, thereby leading to a smaller tunneling probability overall.^[^ ^42^ ^]^ In addition, side‐chain‐dependent and pH‐dependent conductivity of peptides were observed.^[^ ^40^ ^]^ Apart from the tunneling current, length‐dependent electrical properties were also observed in blockade currents through nanopore testing (Figure 3c,d).^[^ ^43^ ^]^ It was found that peptides with only one amino acid difference in length could be differentiated by the amplitudes and durations of blockade currents, suggesting that each amino acid contributes individually to the current blockade. The quantitative results offer a means to evaluate the size of peptides.

Building relationships between electrical characteristics, structure, and function for conformationally constrained peptides is necessary to study the complicated structures and functions of proteins using single‐molecule electrical testing techniques. It has been observed that, while measuring single‐molecule conductance on an MCBJ setup, the configurations of tetrapeptides (4 Ala) may adjust to different metastable structures, some of which could exhibit higher conductivity than dipeptides (2 Ala), suggesting that non‐linear configurations might be more beneficial for charge transport.^[^ ^44^ ^]^ In general, the formation of a higher‐order structure for the shorter peptides is unfavorable because the enthalpy gain from forming a small number of intramolecular hydrogen bonds cannot adequately compensate for the large entropy loss. For long peptides, the α‐helix structure is the most prevalent secondary structure in proteins in specific pH ranges.^[^ ^45^ ^]^ The helical structure has attracted much attention because its parallel assemblies are a universal motif found in biological electron‐transfer systems.^[^ ^46^ ^]^ Compared with stretched peptides, charge transport through the α‐helix peptides is more efficient over long distances, this is attributed to the dominance of the hopping mechanism, wherein amide groups serve as the hopping sites (Figure 3e).^[^ ^42^, ^47^ ^]^ Considering the important role of thermal structural fluctuations in biological activities and the fact that each amino acid backbone can rotate quite facilely over large angles, the charge transport behavior of a peptide is thus expected to exhibit a strong dependence on the Ramachandran angles (Ψ and Φ) (Figure 3f).^[^ ^48^ ^]^ Hence, a two‐step model in charge transport has been proposed: rotation of the backbone (100–150 fs), and then the charge transport. At an extreme deflection of the Ramachandran angle, where two neighboring carbonyl groups are separated by a distance of only ≈ 2.8 Å, electron coupling between them is found to be very strong, and the charge transport barrier becomes negligible compared to that observed at the average angle (0.4 eV). Subsequently, the induced amide radical cation, as stabilized by neighboring amide groups, can hop among the amide sites with a rate constant of ≈ 10^10^ s^−1^.^[^ ^49^ ^]^ This idea is further reinforced when bridging adjacent consecutive turns in a helical peptide. In this case, the charge transport changes from hopping to tunneling through the β‐strand, due to the reduction of vibrational fluctuations and destructive quantum interference between the side‐bridge and the backbone (Figure 3g).^[^ ^50^ ^]^

Life is usually homochiral, and all biological systems predominantly use left‐handed amino acids and right‐handed sugars. When an electron moves through a chiral molecule, the probability of electron transmission presents distinct chirality dependence, known as chirality‐induced spin selectivity (CISS).^[^ ^51^ ^]^ The CISS effect has been demonstrated by electron transportation through chiral biomolecule layer(s), such as DNA and Bacteriorhodopsin.^[^ ^51^, ^52^ ^]^ STM‐BJ, on the other hand, provides a direct and reliable method to evaluate the CISS effect manifesting as the conductance magnitude (Figure 3h).^[^ ^53^ ^]^ The ratio in current between the two spin states is ≈ 10 (at 0.7 V) and the charge transport barrier is ≈0.5 eV, indicating the high efficiency of peptides in spin filtering.^[^ ^54^ ^]^ These facts suggest a connection between electromagnetic field effects with molecular chirality in living organisms.

## 4 Single‐Molecule Analysis of Proteins

### 4.1 Nanopore‐Based Protein Sequencing

Biomacromolecules, such as nucleic acids and proteins, serve as the storage and transmission media for genetic information within living organisms. Unlike the commercially available and extensively applied DNA sequencing technologies, protein sequencing currently only has a very small commercial scale due to high costs and immaturity in technology. It is indeed more challenging for protein sequencing, given the need to distinguish among 20 distinctive amino acids and the complexity of steric configurations in proteins. In addition, the amplification of protein is not as straightforward as DNA duplication.^[^ ^55^ ^]^ Currently, the mainstream protein sequencing techniques are still Edman degradation and mass spectrometry, which have been developed since the 1950s and 1980s.^[^ ^56^ ^]^ However, both techniques fall short of DNA sequencing methods in terms of accuracy, efficiency, and affordability. For instance, Edman degradation is very slow (one cycle takes ≈ 1 h) and is limited by the protein length (<30 AA by the cyclical derivatization), while mass spectrometry is limited by dynamic range, concentration, molecular weight, and pretreatment.^[^ ^57^ ^]^ In particular, both methods are not capable of working on a complete sequence, but rather on degraded fragments. The great success of nanopore techniques in nucleic acid sequencing has prompted further exploration in protein sequencing.^[^ ^58^ ^]^ Typically, in nanopore‐based sequencing, the protein should be unfolded via denaturant treatments before translocation through the nanopore channel, and then the moving speed of the protein should be controlled to allow for sufficient detection of each amino acid. This can be realized either by selecting a proper solvent medium,^[^ ^59^ ^]^ modifying the nanopore surfaces,^[^ ^60^ ^]^ or by using molecular motors such as DNA motor proteins.^[^ ^61^ ^]^

**Figure 4:**
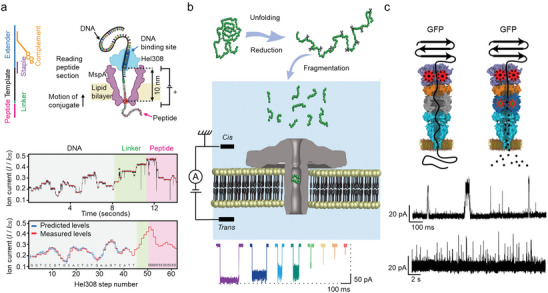
Protein sequencing based on a nanopore. a) Schematic of peptide sequencing with the assistance of DNA helicase using a nanopore (upper), along with the presentation of typical ion current signals during the test (lower). Reproduced with permission.^[^ ^62^ ^]^ Copyright 2021, American Association for the Advancement of Science. b) Schematic of protein identification based on the fingerprint of polypeptide fragments. Reproduced with permission.^[^ ^63^ ^]^ Copyright 2022, American Chemical Society. c) Schematic of controlled translocation of protein through an integrated multiprotein complex. Reproduced with permission.^[^ ^64^ ^]^ Copyright 2021, Springer Nature.

In a recent pivotal work, a DNA helicase was used as a motor enzyme to pull the peptide transport through the nanopore in single amino acid steps, and a captured, unique ladder‐like current signal was accurately identified (**Figure** **4**a).^[^ ^62^ ^]^ Furthermore, the helicase allows a “rewind” read of the peptide, which can significantly reduce sequencing errors. Owning to the ingenious designs, peptide variants with single amino acid substitutions were successfully identified as well, and the average accuracy of identifying amino acid variants was calculated to be 87%. In addition to the stepwise amino acid reading, another strategy is to cut the protein into polypeptide fragments first and compare the signals obtained from these blocks with the database of known proteins (Figure 4b).^[^ ^63^ ^]^ It should be noted that this method relies on the establishment of large databases and is limited to known protein units. Recently, a proteasome‐nanopore was prepared in a bottom‐up design, with two approaches to realize single‐molecule protein sequencing (Figure 4c).^[^ ^64^ ^]^ The first is “thread and read”, in which a protease with strong unfolding activity unfolds the target protein, and the stretched peptides are then driven through the nanopore by an electroosmotic flow. The second is “chop and drop”, in which the proteasome cuts the unfolded protein, and the peptides are recognized by the nanopore during the translocation process. These two sequencing approaches guarantee the performance of this multiprotein molecular machine for sequencing.

### 4.2 Structure and Shape of Proteins

Proteins fold into various tertiary structures, and the identification and regulation of the protein structure are indispensable in the pursuit of protein analyses. Unlike the STM‐BJ technique described above, the STM imaging technique allows for the visualization of the spatial structure of proteins and examines their electronic properties directly within their natural environments. The magnitude of the tunneling current during tip scanning is directly related to the separation between the tip and the substrate, indicating variations in surface topography.^[^ ^65^ ^]^ Cytochrome *b*_562_, modified through genetic engineering to include cysteine residues capable of binding to the Au surface in an STM setup with two controlled molecular orientations, exhibits significantly higher conductance compared to synthetic molecules of similar dimensions.^[^ ^66^ ^]^ The heme is located asymmetrically in the interior of the protein relative to the overall topology, the two orientations result in different distances between the redox‐active heme site and the electrodes, and consequently different molecular conductance.^[^ ^66^, ^67^ ^]^

**Figure 5:**
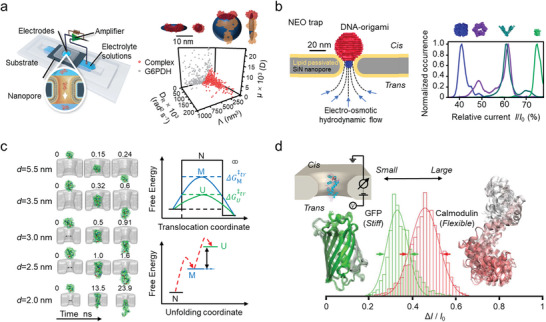
Electrical characterizations of protein spatial structure and structural change with nanopore. a) Schematic of single protein identification through a bilayer‐coated nanopore (left), and the characterizations of single protein and protein complex (right). Reproduced with permission.^[^ ^68^ ^]^ Copyright 2016, Springer Nature. b) Schematic of Nanopore electro‐osmotic trap (NEO trap, left), and the mass‐ and shape‐dependent single protein identification (right). Reproduced with permission.^[^ ^23d^ ^]^ Copyright 2021, Springer Nature. c) Conformationally excited states of protein during translocation, and schematic energy diagram for translocation. Reproduced with permission.^[^ ^23c^ ^]^ Copyright 2021, National Academy of Sciences, U.S.A. d) Protein size and structural fluctuations measurements using solid‐state nanopore. Reproduced with permission.^[^ ^70^ ^]^ Copyright 2017, American Chemical Society.

In addition to STM techniques, nanopore sensors can also provide valuable in situ information about protein morphology relying on electrical signals (**Figure** **5**a).^[^ ^68^ ^]^ Specifically, the rotational motion of a single non‐spherical protein within a long cylindrical nanopore channel could induce discernible current fluctuations and present an extreme ratio between the current blockage in their crosswise versus lengthwise direction. Using the maximum Δ*I* value, the shapes of ten different proteins have been estimated. Meanwhile, the potential of the nanopores in distinguishing the volume, charge, dipole moment, and rotational diffusion coefficient of the protein was also demonstrated. In another ingenious design, a nanopore electro‐osmotic trap (NEOtrap) was constructed by docking a ball‐shaped DNA origami structure over one of the entrances of a solid nanopore (Figure 5b).^[^ ^23d^ ^]^ The NEOtrap was then proved to be able to distinguish different proteins, including their mass, type, and shape, based on their characteristic peaks in the blockade current histogram. These research findings show that single‐molecule current signals can provide valuable information regarding the dynamic structures of the biomolecules.

Channels and pores that respond directly to molecules or physical stimuli are widely found within life systems.^[^ ^69^ ^]^ The translocation of protein across channels and pores is important both in fundamental science and biotechnology. Nanopores have been used to detect protein fluctuations and conformational changes during translocation. The translocation behavior of the folded cytochrome c (cyt c, with a diameter of ≈3 nm) through ultra‐thin SiN solid‐state nanopores with diameter ranging from 1.5 to 5.5 nm shows that a protein molecule can pass through a narrower channel compared with the diameter of the protein in its folded state (Figure 5c).^[^ ^23c^ ^]^ When the diameter of the nanopore (3–5.5 nm) is larger than that of cyt c, cyt c would transport freely with a short‐lived blockade current. When the nanopore is narrowed to a comparable size, such as 2.5 nm, cyt c could pass through the pore after unfolding the residual α‐helices which is assisted by the external electric field. When the pore size is further decreased to 2.0 nm or even less, translocation of cyt c would only occur after it is fully unfolded. Furthermore, nanopores can be used to study protein transformation in its native state. For example, the conformation of the calcium‐binding protein, calmodulin, becomes stretched and flexible upon calcium binding, and the current signal caused by calcium‐bound calmodulin shows deeper blockade amplitudes and longer dwell times than that of the unbound calmodulin (Figure 5d).^[^ ^70^ ^]^ This indicates that nanopores hold promise for assessing the ligand‐binding events of proteins.

### 4.3 Charge Transport of Proteins

**Figure 6:**
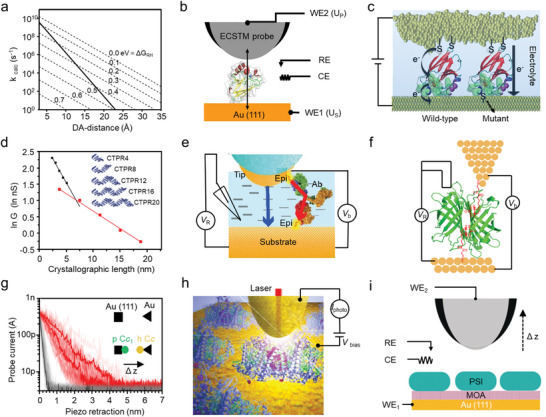
Charge transport through single proteins. a) Relationship between the donor‐accepter distance (DA‐distance) and the reaction rate constant for single‐step and two‐step electron tunneling transfer. Reproduced with permission.^[^ ^73^ ^]^ Copyright 2005, National Academy of Sciences, U.S.A. b) Schematic of conductance measurements of azurin using electrochemical scanning tunneling microscopy break junctions. Reproduced with permission.^[^ ^76^ ^]^ Copyright 2011, American Chemical Society. c) Schematic of charge transport through wild‐type azurin and its mutant. Reproduced with permission.^[^ ^79^ ^]^ Copyright 2017, American Chemical Society. d) Conductance of a series of modular proteins of varying length. Reproduced with permission.^[^ ^81^ ^]^ Copyright 2022, American Chemical Society. e) Schematic of long‐distance charge transport through proteins without electrochemical activity. Reproduced under the terms of the CC‐BY‐NC‐ND license.^[^ ^82^ ^]^ Copyright 2019, The Authors, published by PNAS. f) Schematic of charge transport through streptavidin protein bound to thiolated biotin‐treated electrodes. Reproduced with permission.^[^ ^84^ ^]^ Copyright 2020, American Chemical Society. g) Conductance measurements of a protein complex (human cytochrome c (hCc) and plant cytochrome Cc1 (pCc1)). Reproduced under the terms of the CC‐BY license.^[^ ^85^ ^]^ Copyright 2018, The Authors, published by Springer Nature. h) Schematics of photocurrent measurements and i) conductance measurements of photosystem I. h) Reproduced with permission.^[^ ^86^ ^]^ Copyright 2012, Springer Nature. i) Reproduced with permission.^[^ ^87^ ^]^ Copyright 2019, Wiley‐VCH Verlag GmbH & Co. KGaA, Weinheim.

Nature has developed highly specialized protein systems in organisms with high charge transport efficiency at long distances, such as in aerobic respiration and photosynthesis, which play a key role in the energy conversion and transfer processes and make life possible on the earth.^[^ ^71^ ^]^ Generally, the charge transport duration should be within the range of milliseconds to microseconds to make them function properly for biological redox machines. However, considering the size of these proteins (2–7 nm) and their chemical composition (poorly conductive amino acids), well‐controlled and highly efficient charge transport is probably the most important puzzle in the biological system. Especially, these events highly rely on well‐defined biochemical structures.^[^ ^72^ ^]^ If it is in the tunneling regime, the maximum center‐to‐center distance of charge transport should be no >20 Å. However, in some cases, it can occur at a speed far exceeding this direct tunneling limit, e.g. through redox enzyme assemblies.^[^ ^73^ ^]^ One possible mechanism for such long‐distance transport is the hopping mechanism. According to classical (Marcus) theory, the activation barrier for an adiabatic charge transport depends on two parameters, the driving force (−Δ*G* ^0^) and the reorganization energy (λ).^[^ ^74^ ^]^ The protein folding plays a central role in lowering λ in their native environments. For example, in a two‐step hopping event with λ = 0.8 eV and a decay constant (β) of 1.1 Å^−1^, distance dependence of charge transport rates of tunneling and hopping when Δ$G_{\mathrm{GH}}^{0}$ = −Δ$G_{\mathrm{HP}}^{0}$ and r_RH_ = r_HP_ (R, H, and P denote the reactant, intermediate, and product, respectively) are shown in **Figure** **6**a. With a hopping mechanism, charge transport crossing a distance of 20 Å can happen in sub‐milliseconds, which is 10^4^ times faster than that with a tunneling mechanism. Hopping can facilitate the charge transport distances of over 20 Å. Meanwhile, the free‐energy changes for endergonic intermediate steps are no >0.2 eV. This model is consistent with the fact that the characteristic λ for redox charge transport is at 0.6–0.8 eV to facilitate high charge transport.^[^ ^73^ ^]^

Redox proteins play an important role in biological charge transport and have potential applications in bioelectronics. For these proteins, their conductance can reach a maximum value when electrons are injected above a certain redox potential. Azurin is a blue single‐copper protein that functions as a soluble electron carrier physiologically associated with oxidative stress responses by switching between its redox states (Cu^I/II^), and it is also a long‐standing and convenient system to investigate the charge transport through protein.^[^ ^71^, ^75^ ^]^ By means of electrochemical scanning tunneling microscopy break junction (ECSTM‐BJ), decay constants of 2.9 ± 0.2 nm^−1^ and 4.5 ± 0.3 nm^−1^ were obtained, respectively, for the oxidized state and the reduced state of azurin, indicating that the charge transport is dominated by a hopping mechanism (Figure 6b).^[^ ^76^ ^]^ It is also found that the negative probe potential is not favorable for charge injection into the reduced azurin, which presents a low‐conductance state. In contrast, the charge injection for the oxidized azurin is much more efficient.^[^ ^77^ ^]^ A transition voltage at 0.4 V was obtained when there was no physical contact between the probe and the protein, while an ultralow transition voltage at −0.06 V was observed when physical contact was established.^[^ ^78^ ^]^ Considering the biological function of azurin, it can be speculated that redox‐dependent decay constant and conductance, as well as asymmetric coupling, could control the charge transfer rate and direction along the respiratory chains without minimum energy loss. In other words, charge storage occurs in the weak coupling contact, and charge transfer is favored with strong coupling in biosystems. In addition, charge transport in the single‐protein junction relies significantly on the protein structure. Introducing a slight structural modification, such as changing from a wild‐type redox protein to a single point mutant (K41C), could turn the charge transport mechanism from hopping, which was found in the wild type, to coherent tunneling (Figure 6c).^[^ ^79^ ^]^

The research based on STM‐BJ shows that some non‐redox proteins are also good electronic conductors and have the potential to work as molecular transistors.^[^ ^80^ ^]^ A small decay constant was observed for the consensus tetratricopeptide repeat proteins, in which the conductance even exceeds that of the canonical molecular wire oligo(phenylene–ethylenene) after a certain length (over 6 nm) (Figure 6d).^[^ ^81^ ^]^ The good conductivity, based on a hopping mechanism, was also observed in six proteins lacking known electrochemical activity with at least one good contact (ligand‐receptor interactions) (Figure 6e).^[^ ^82^ ^]^ Significantly, the conductance of a single Fab fragment (2.5 nm) is much smaller than that of an antibody (4.5 nm).^[^ ^83^ ^]^ In ECSTM‐BJ measurements, the maximum conductance of three non‐redox proteins was found at very close potentials (300 mV vs NHE) (Figure 6f).^[^ ^84^ ^]^ This indicates that the charge transport might follow the same mechanism, arising from the common intrinsic feature of these proteins. For the non‐redox proteins, the folded structure must have played an important role, i.e., a similar potential dependence may be a result of the reduction of the reorganization energy barrier inside the protein.^[^ ^82^ ^]^ The weak distance dependence and high intrinsic conductance of non‐redox proteins consistently indicate that once injected, charges could move readily at a long range within the interior of the protein, and such behavior could be studied through single‐molecule electronic devices with an electrochemical gate.^[^ ^84^ ^]^

In the 50 mm sodium phosphate buffer solution, a minimal decay constant of 0.5 ± 0.3 nm^−1^ was found between reduced plant cytochrome Cc1 subunit (pCc1) and oxidized human cytochrome c (Cc), allowing long‐distance charge transport beyond 10 nm (Figure 6g).^[^ ^85^ ^]^ Because of the significantly reduced cationic concentration at the protein interface, water molecules are regarded as transport mediators in the constrained aqueous interface between proteins. In addition, the charge transport presents a marked potential dependence. The sensitive potential‐dependent charge transport indicates a plausible biological mechanism in aqueous solution. Photosynthesis is an essential process for plants, algae, and bacteria, as it converts light energy, CO_2_, and water into carbohydrate molecules. The photosystem I (PS I) complex is a light‐driven oxidoreductase, in which photoexcited electrons could transfer along the reaction‐center electron transfer chain with an internal quantum efficiency close to 1. When the cysteine mutational PS I was illuminated by 633 nm laser light with a power of 4 mW, an intriguingly large photocurrent of ≈10 pA with a turnover time of ≈16 ns was obtained through scanning near‐field optical microscope in an ultrahigh‐vacuum environment at room temperature (Figure 6h).^[^ ^86^ ^]^ By changing the distance between the PS I and the probe in solution, a much lower decay constant (β < 4 nm^−1^) was obtained with the sample potential at 200–400 mV (Figure 6i).^[^ ^87^ ^]^ These results prove that PS I, functioning as electron collection or hole injection, supports electrochemically gated long‐distance charge transport. The charged sites in PS I, electrostatic interactions‐induced reduction of ionic concentration, and water reorganization energy should be responsible for the low decay constant.

### 4.4 Single‐Molecule Detection of Biological Interactions and Reactions

**Figure 7:**
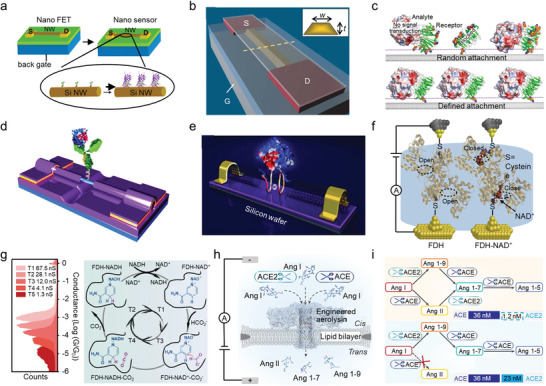
Electrical detection of biological interactions and reactions. a) Schematic representation of streptavidin binding to a SiNW FET through the biotin‐streptavidin interaction. Reproduced with permission.^[^ ^16c^ ^]^ Copyright 2001, The American Association for the Advancement of Science. b) CMOS‐compatible FET fabrication via anisotropic wet etch. Reproduced with permission.^[^ ^91^ ^]^ Copyright 2007, Springer Nature. c) Schematic of a single antibody‐decorated SiNW FET. Reproduced under the terms of the CC‐BY license.^[^ ^93^ ^]^ Copyright 2021, The Authors, published by Wiley‐VCH GmbH. d) Schematic of orientation‐controlled binding of a protein on the SWCNT surface. Reproduced with permission.^[^ ^95^ ^]^ Copyright 2014, WILEY‐VCH Verlag GmbH & Co. KGaA, Weinheim. e) Schematic of a single protein junction with a DNA aptamer on SWCNT electrodes. Reproduced with permission.^[^ ^96^ ^]^ Copyright 2011, WILEY‐VCH Verlag GmbH & Co. KGaA, Weinheim. f) Schematic of charge transport in a single‐molecule FDH junction and an FDH‐NAD^+^ junction. Reproduced under the terms of the CC‐BY‐NC‐ND license.^[^ ^98^ ^]^ Copyright 2020, The Authors, published by Elsevier. g) Conductance changes of the corresponding process of an enzymatic reaction with a single‐molecule FDH. Reproduced with permission.^[^ ^99^ ^]^ Copyright 2023, Springer Nature. h) Identification of degradation products upon Ang I cleavage by ACE and ACE2. i) Degradation pathway of Ang I in the presence of ACE and ACE2. Reproduced with permission.^[^ ^100^ ^]^ Copyright 2023, Springer Nature.

Real‐time, label‐free, and sensitive detection of biomolecules and biological processes at the single‐molecule level could provide substantial information for disease diagnosis and treatment, drug discovery, and biological process visualization. The concentration of the majority of proteins important in disease diagnosis ranges from 10^−16^ to 10^−12^ m in serum.^[^ ^88^ ^]^ Single‐molecule FET has been successfully applied to very dilute analyte samples with high sensitivity, for example, ion detection with single‐charge resolution.^[^ ^89^ ^]^ With a biotin‐modified SiNW‐FET, the detection limit of streptavidin could be down to the picomolar scale, and good linearity was obtained between the conductance and the monoclonal antibiotin concentration below ≈10 nm (**Figure** **7**a).^[^ ^16c^ ^]^ With a SiNW‐FET array design, multiplexed detection was achieved in an antibiotin sample with a concentration of only 0.9 pg·mL^−1^.^[^ ^90^ ^]^ Employing a top‐down strategy, a complementary metal oxide semiconductor (CMOS) FET was developed by anisotropic wet etching of commercially available (100) silicon‐on‐insulator wafers (Figure 7b). The resulting CMOS FET has a high sensitivity to specific label‐free antibodies, with a detection limit as low as 100 fM, and can be used for real‐time monitoring of the cellular immune response.^[^ ^91^ ^]^ Likewise, the dynamic desorption and adsorption of a single ATPase on a chemically functionalized SiNW surface have been successfully detected using a similar setup.^[^ ^92^ ^]^ To achieve a site‐specific connection with a favorable orientation at the surface of SWCNTs to avoid functional differences between devices, a surface‐controlled strategy was developed (Figure 7c).^[^ ^93^ ^]^ Firstly, a noncanonical amino acid (ncAA) modified‐capture protein was modified to the sidewalls of SWCNT via click chemistry. Then, lactamase was captured in a controlled manner, and the concentration‐dependent current changes were observed. Modulating λ_D_ and changing ncAA positions in the protein revealed that the electrostatic gating effect is mainly determined by the local electrostatic property within λ_D_, rather than the net charge of captured proteins. On these devices, where samples are prepared at very low concentrations, the electrical response still inevitably shows some concentration dependence due to the presence of more than one active sensing center on a single SiNW or SWCNT.^[^ ^94^ ^]^ In the following research, a real single‐molecule sensitivity detection, i.e., single point decoration of SiNW‐FET, has been achieved (Figure 7d).^[^ ^95^ ^]^ Specifically, employing a combination of electron beam lithography and chemical etching, a 30 nm hydrogen‐terminated nanogap is opened on the surface of the SiNW, a size only sufficient to be modified with a single (or at most two) H1N1 antibody at a time. Upon the addition of H1N1, large‐amplitude two‐level fluctuations with a conductance difference of 3 ns were observed even with a trace amount of 7.8 pg·mL^−1^. The reversible change in electrostatic potential is attributed to the antibody‐antigen association/dissociation.

The single‐molecule junctions and nanopore techniques also show increasing potential in biological interactions and reaction detection. For example, a device was prepared by inserting a single DNA aptamer into the SWCNT gap, in which the G4 conformation of the aptamer binds to thrombin with high specificity and affinity (Figure 7e).^[^ ^96^ ^]^ Upon the addition of thrombin, the resistance presented a drastic reduction by one order of magnitude, indicating the enhanced charge transport of G4. Formate dehydrogenase (FDH) is an important oxidoreductase that catalyzes the dehydrogenation of formate to form carbon dioxide through the electric coupling between FDH and nicotinamide adenine dinucleotide (NAD^+^).^[^ ^97^ ^]^ Using STM‐BJ, charge transport through single active FDH systems shows that the insertion of NAD^+^ into the FDH active site results in a 21‐fold increase in conductance as a result of the through‐bond charge transmission path (Figure 7f).^[^ ^98^ ^]^ This allows the reaction states to be distinguished based on their characteristic conductance and leads to the finding that the enzyme reaction does not strictly follow the widely accepted Theorell–Chance mechanism. Instead, the apoenzyme state could be bypassed (Figure 7g).^[^ ^99^ ^]^ The high sensibility of nanopores also makes it available for biological reaction detection. The enzyme crosstalk effect between angiotensin‐converting enzyme (ACE) and angiotensin‐converting enzyme 2 (ACE2) on the renin–angiotensin system (RAS) is closely related to the biological functions of RAS, but the quantitative study is challenging due to the complexity and short half‐life of the substrates. With a biological nanopore, the products of multiple angiotensin peptides (Ang) have been monitored in real‐time, and the results indicated that ACE2 could selectively inhibit ACE from cleaving Ang I even at a much lower concentration (Figure 7h,i).^[^ ^100^ ^]^

In biosystems, the demands for effective information transfer between proteins are conflicting. For example, in electron transfer chains, tight binding is desired to produce high rates, while binding should be sufficiently weak to allow a high turnover rate and overall efficiency.^[^ ^85^ ^]^ These demands result in a stepwise association process between the protein partners. The intermolecular charge transfer of proteins and protein complexes that takes place in physiological environments is a more complex situation due to various configurations of proteins and environmental disturbances.^[^ ^76^ ^]^ Instead of a snapshot of the final state, real‐time monitoring of the ultra‐fast dynamic process, such as conformational change, is of great importance in mechanistic studies.^[^ ^101^ ^]^

**Figure 8:**
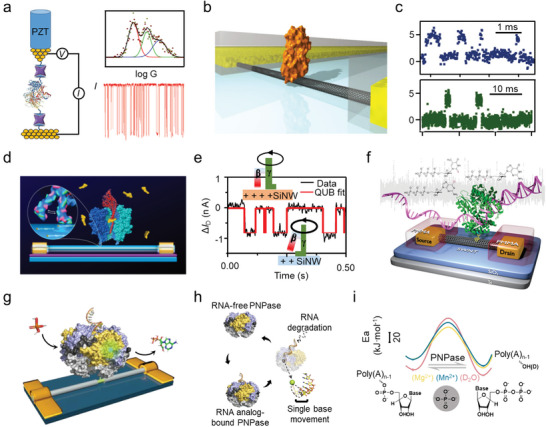
Electrical detection of dynamics bioreaction processes. a) Monitoring of the enzyme activity of Φ29 polymerase using a thiolated biotin‐functionalized STM‐BJ. Reproduced with permission.^[^ ^102^ ^]^ Copyright 2019, American Chemical Society. b) Monitoring of the lysozyme activity using a SWCNT FET. c) Two‐level current fluctuations of lysozyme during substrate hydrolysis. Reproduced with permission.^[^ ^104^ ^]^ Copyright 2012, American Association for the Advancement of Science. d) Monitoring of the F1‐ATPase activity via a SiNW FET. e) Continuous recording of currents over time during F1 hydrolysis. Reproduced with permission.^[^ ^107^ ^]^ Copyright 2017, American Chemical Society. f) Monitoring of the DNA polymerase I activity via a SWCNT FET. Reproduced with permission.^[^ ^108b^ ^]^ Copyright 2015, American Chemical Society. g) Schematic of a SiNW FET device decorated with a single PNPase molecule. h) Working principle of PNPase. i) Energy barrier in the hydrolysis of the phosphodiester bond. Reproduced under the terms of the CC‐BY license.^[^ ^109^ ^]^ Copyright 2023, The authors, published by Springer Nature.

On an STM setup, a Φ29 polymerase was linked to the electrode through biotin‐streptavidin recognition, allowing for the real‐time monitoring of configuration change‐induced electrical disturbances during enzymatic reactions (**Figure** **8**a).^[^ ^102^ ^]^ The results show that enzymatic reactions with Φ29 polymerase are accompanied by rapid (ms level) current fluctuations, which have a significant difference of more than two‐fold when switching between the open and closed conformations. Although the fabrication of single‐molecule FET devices is more complicated compared to STM‐BJ devices. Taking lysozyme as an example, its configurational change (open and closed conformations) and catalytic activities have been successfully studied on a SWCNT‐FET by monitoring the current fluctuations over time.^[^ ^103^ ^]^ A continuous measurement of 10 min revealed that lysozyme is a processive enzyme, and the conductance presents a two‐level random fluctuation during the hydrolysis of glycosidic (Figure 8b).^[^ ^104^ ^]^ The slow fluctuation of low currents originates from the catalytic switching events, i.e., the processive hydrolyzation of chemical bonds at a rate of 15 Hz, whereas the fast fluctuation of high currents originates from a non‐productive hinge motion at 330 Hz (Figure 8c). The pH dependence of lysozyme proved to be raised from the nonproductive rapid motions and an inactive closed conformation. Further research reveals that lysozyme spends 88% of the total time processing the linear substrate on a 600 s time scale. However, the proportion reduces to 51% for cross–linked peptidoglycan, with the catalytic rate changing from 35.9 ± 17.6 s^−1^ for the linear substrate to 30.0 ± 14.5 s^−1^ for the cross–linked substrate.^[^ ^105^ ^]^

Connecting the catalytic domain of a cAMP‐dependent protein kinase A (PKA) to the SWCNT‐FET, three‐level *I*_sd_(t) fluctuations were observed in the presence of ATP and can be attributed to three different states, i.e., binding of substrate, binding of ATP, and the formation of the ternary complex, which has complete catalytic function, respectively.^[^ ^106^ ^]^ Furthermore, the kinetics parameters of each event can be measured accordingly through a SiNW‐FET device (Figure 8d).^[^ ^107^ ^]^ The high current state presents a single exponential behavior with longer average dwell times (≈237.44 ms), which is attributed to the ATP‐binding state (Figure 8e). In contrast, the low current state is biexponential, corresponding to a sequential two‐step reaction: ATP hydrolysis and Pi release. A hydrolysis rate of 1.69 × 10^8^ m ^−1^ s^−1^ at 20 °C is obtained from statistical analysis.

Replication and repair processes involved in the proteases DNA polymerases are required for all life. Apart from thermostability and specificity, fidelity is also a characteristic of DNA polymerases, especially for certain enzymes. Kinetics studies at single‐nucleotide resolution have been carried out with SWCNT‐FETs. It is observed that each base incorporation presents a similar current change for both native dNTPs and analogs, indicating that the incorporation rate is mainly dominated by molecular recognition rather than the covalent‐bond formation process (Figure 8f).^[^ ^108^ ^]^ The high incorporation sensitivity is attributed to the difference in recognition ability between the native and analog substrates, and the enzyme applies a dynamic stability‐checking mechanism for each nascent base pair.^[^ ^108b^ ^]^ RNA degradation dynamics, including RNA analog binding, single‐nucleotide hydrolysis, and single‐base movement, with single‐base resolution are also achieved on a nucleic acid exonuclease (polynucleotide phosphorylase, PNPase) by means of SiNW‐FETs (Figure 8g–i).^[^ ^109^ ^]^ The addition of RNA analogs results in an increase in current due to the expansion of the PNPase structure. The occurrence proportion of the PNPase‐free structure gradually decreases with increasing temperature, while the occurrence proportion of the RNA analog‐bound structure and stability gradually increases. Upon addition of phosphoric acid, a three‐level conductance fluctuation appears, corresponding to the RNA analog degradation structure, RNA analog‐bound structure, and the PNPase free structure transitioning from a high state to a low state, respectively. Similar to the case of PNPase, another interesting example is to integrate an individual disordered c‐Myc bHLH‐LZ domain, a typical Intrinsically disordered protein, into SiNW‐FETs, therefore enabling label‐free, in situ, and long‐term measurements of the self‐folding/unfolding process of c‐Myc and revealing its interaction mechanism with Max and inhibitors.^[^ ^14h^ ^]^

## 5 Conclusion and Outlook

In this review, the current mainstream single‐molecule electrical detection techniques, which find diverse applications in the research of protein structures and functions, are introduced. Single‐molecule junctions are mainly employed to investigate the charge transport characteristics of single molecules ranging from amino acids to protein complexes by conductance measurements. Studies indicate that the conductivity of peptides is highly contingent on their spatial structures. Folded peptides, like α chains, generally demonstrate better conductivity than those with the stretched configuration. The latter can even exhibit poorer conductivity than saturated alkane chains. The significant contrast in conductivity is believed to arise from different charge transport mechanisms, such as tunneling and hopping. Nanopore techniques have shown promising progress in contributing to the sequencing of peptides and proteins. A recent breakthrough is the use of a DNA motor protein alongside the biological nanopore to regulate the translocation speed of proteins through the nanopore, significantly enhancing the accuracy and resolution of this method. Apart from reading amino acids along the peptide chain, based on the relative blockage currents and dwell times, nanopores have been used to estimate the shape and dimensions of a protein. Single‐molecule field‐effect transistors, by allowing continuous measurements at a fixed molecular junction, offer distinctive advantages for the exploration of biological processes and biomolecule recognition, such as monitoring enzyme reactions in biological solutions in real‐time through the fluctuations in channel current. In summary, these cutting‐edge single‐molecule electrical detection techniques not only deepen our understanding of protein structures and functions but also have the potential to drive significant progress in diagnostics and drug development.

The spatial structures of proteins and peptides, built on non‐covalent interactions, are highly dynamic, with configurations interconverting at a timescale ranging from milliseconds to sub‐microseconds. In tandem, many chemical reactions also occur within the millisecond to microsecond range. Hence, there is a demand for testing methods with high temporal resolution (ns–µs) to capture these events and their constituent sub‐events accurately. Beyond this, there is a growing expectation for multimodal characterization platforms that integrate electrical, optical, and mechanical testing techniques.^[^ ^110^ ^]^ These platforms are anticipated to offer more precise and comprehensive insights into studying the configurations, properties, and functions of proteins, especially in their natural biomacromolecular environment. Lastly, from a technical development standpoint, molecular device miniaturization based on the standard CMOS technology and sensitive detection of diverse biomolecules by modularized devices are continuing pursuits.^[^ ^111^ ^]^

## Conflict of Interest

The authors declare no conflict of interest.
